# Proteomic profile data of *Klebsormidium nitens* alga grown in control and saline conditions

**DOI:** 10.1016/j.dib.2025.112335

**Published:** 2025-11-28

**Authors:** Alix Martinet, Carole Pichereaux, David Wendehenne, Sylvain Jeandroz, Valérie Nicolas-Francès

**Affiliations:** a Université Bourgogne Europe, Institut Agro Dijon, INRAE, UMR Agroécologie, 17 rue Sully, 21065 Dijon cedex, France; b Fédération de Recherche (FR3450), Agrobiosciences, Interactions et Biodiversité (FRAIB), Université de Toulouse, CNRS, Université Toulouse (UT), 24 Chemin de Borderouge, Campus INRAE 31326 Castanet-Tolosan cedex, France; c Institut de Pharmacologie et de Biologie Structurale (IPBS), Université de Toulouse, CNRS, Université de Toulouse (UT), 205 route de Narbonne, 31400 Toulouse, France; d Infrastructure Nationale de Protéomique, ProFI, UAR 2048, 205 route de Narbonne, 31400 Toulouse, France

**Keywords:** *Klebsormidium nitens*, Proteome, LC-MS/MS, Alga, NaCl stress

## Abstract

*Klebsormidium nitens (K. nitens)* is an alga of the charophyte class used as a model for studying the adaptation of plants to terrestrial life. Its genome has been completely sequenced and 16,215 protein-coding genes have been predicted. In this study, the proteome of *K. nitens* grown under standard conditions or after salt stress was investigated. A total of 1190 proteins were experimentally confirmed and 922 of them were classified according to their cellular location, molecular or biological function. Of these 922 proteins, 62 and 124 were specifically found in the control and salt-treated samples, respectively. However, no specific function or location could not be assigned on the basis of the primary sequences. A protein–protein interaction network based on the 124 proteins found in saline conditions was constructed using STRING analysis. All the data are accessible and are of interest for phycologists, as well as for evolutionary plant biologists, and provide a foundation for future studies investigating how *K. nitens* responds to salt stress.

Specifications Table

**Table:** 

Subject	Biology
Specific subject area	*Klebsormidium nitens* (alga) proteome analysis under two growth condition ie control condition or salt stress condition (NaCl 500 mM)
Type of data	• Image (.jpeg) • Files .raw: raw LC-MS acquisition files (. raw-available via repository) • Files .dat: database query files via Mascot (available via repository) • Files .xls: results files, available via repository (VN01_NaCl_SP and VN_02_NaCl_SP : 500 mM NaCl condition; VN03_CTRL_SP and VN_04_CTRL_SP : control condition) • Supplementary file1 .xls : Sheet total protein: 922 *Klebsormidium niten*s proteins identified in the two control samples and/or the two NaCl samples and having an orthologue in *Arabidopsis thaliana*; Sheet NaCl specific protein : 124 proteins identified only in the two NaCl samples; Sheet Control specific protein : 62 proteins identified only in the two control samples. GO terms : GO terms associated with the 922 proteins; homemade liquid C medium • Supplementary file2 .xls : protein composition of each of the 12 clusters determined using STRING (identifiers*, A. thaliana* orthologues, protein names)
Data collection	*Klebsormidium nitens* strain SAG 13.91 was grown under light and stirring for 1 or 3 h in a modified liquid C medium with or without 500 mM NaCl. *K. nitens* cells were harvested by vacuum and were immediately frozen. Frozen cells were mechanically ground using glass beads. The cell powder was mixed in a solubilisation buffer and the proteins were precipitated with acetone. After trypsin digestion, peptides mixtures were analysed by nano-LC-MS/MS using nanoRS UHPLC system (Dionex, Amsterdam, The Netherlands) coupled to a Q-Exactive Plus mass spectrometer (Thermo Fisher Scientific, Bremen, Germany). Acquired MS and MSMS data were searched with Mascot (version 2.8.0.1, http://matrixscience.com) against a custom-made database containing all *Klebsormidium nitens* proteins from the UniProtKB database. To enhance the functional annotation of *Klebsormidium nitens* proteins, orthogroups were identified using the software OrthoFinder (version 3.0.1b1). The Gene Ontology (GO) terms associated with *Arabidopsis thaliana* proteins were then transferred to the corresponding *Klebsormidium nitens* homologs within the same orthogroups. The GO hierarchy file, available from the Gene Ontology Ressource was used to map initial GO terms to their most relevant parent terms. This selection was performed manually for subcellular localization categories and semi-automatically for biological process and molecular function categories, using the R package ontology Index
Data source location	Université Bourgogne Europe, Institut Agro Dijon, INRAE, UMR Agroécologie, 17 rue Sully, 21065 Dijon cedex, France Infrastructure Nationale de Protéomique, ProFI, UAR 2048, 205 route de Narbonne, 31400
Data accessibility	Repository name: Proteomic profile data of *Klebsormidium nitens* alga grown in control and saline conditions Data identification number: PXD066259 Direct URL to data: https://www.ebi.ac.uk/pride **Password:** wY1vz8CcewiB
Related research article	none

## 1 Value of the Data

• This work represents a first insight into the proteome of the streptophyte alga *Klebsormidium nitens.*
• The corresponding data can help researchers in confirming the presence of *Klebsormidium nitens* proteins and in completing genomic and transcriptomic analyses.
• Gene Ontology was used to gather the proteins into biological process, molecular function and cellular component categories.
• Data may enable researchers to highlight important candidate proteins to study salt stress-response mechanisms in *Klebsormidium nitens* and their evolution in the green lineage.

## 2 Background

*Klebsormidium nitens (K. nitens)*, a photosynthetic alga in the Klebsormidiophyceae family belonging to the streptophyte clade, is phylogenetically closer to terrestrial plants as compared to chlorophytes (which include model green algae such *Chlorella vulgaris* and *Chlamydomonas reinharditi)* [1]. *K nitens* is a freshwater alga that can also grow in dry environments such as rocks [2]. This alga is particularly resistant to many abiotic stresses [3]. Because of its phylogenetic proximity with embryophytes and its ability to withstand abiotic stresses typical of the terrestrial environment, *K. nitens* is a model for the study of plant adaptation to terrestrial life. Morphologically, *K. nitens* takes the form of an unbranched filament composed of several mononuclear cylindrical cells [4]. The *K. nitens* genome was sequenced in 2014 by Hori et al. [4]. Overall, 16,215 protein-coding genes were annotated from the nuclear and organelle genome sequences.

The aim of our data is to produce an experimental proteome of *K. nitens* under standard culture condition and under salt stress (500 mM NaCl). We anticipate that certain proteins can only be produced in one or the other of these conditions.

## 3 Data Description

**Fig. 1:**
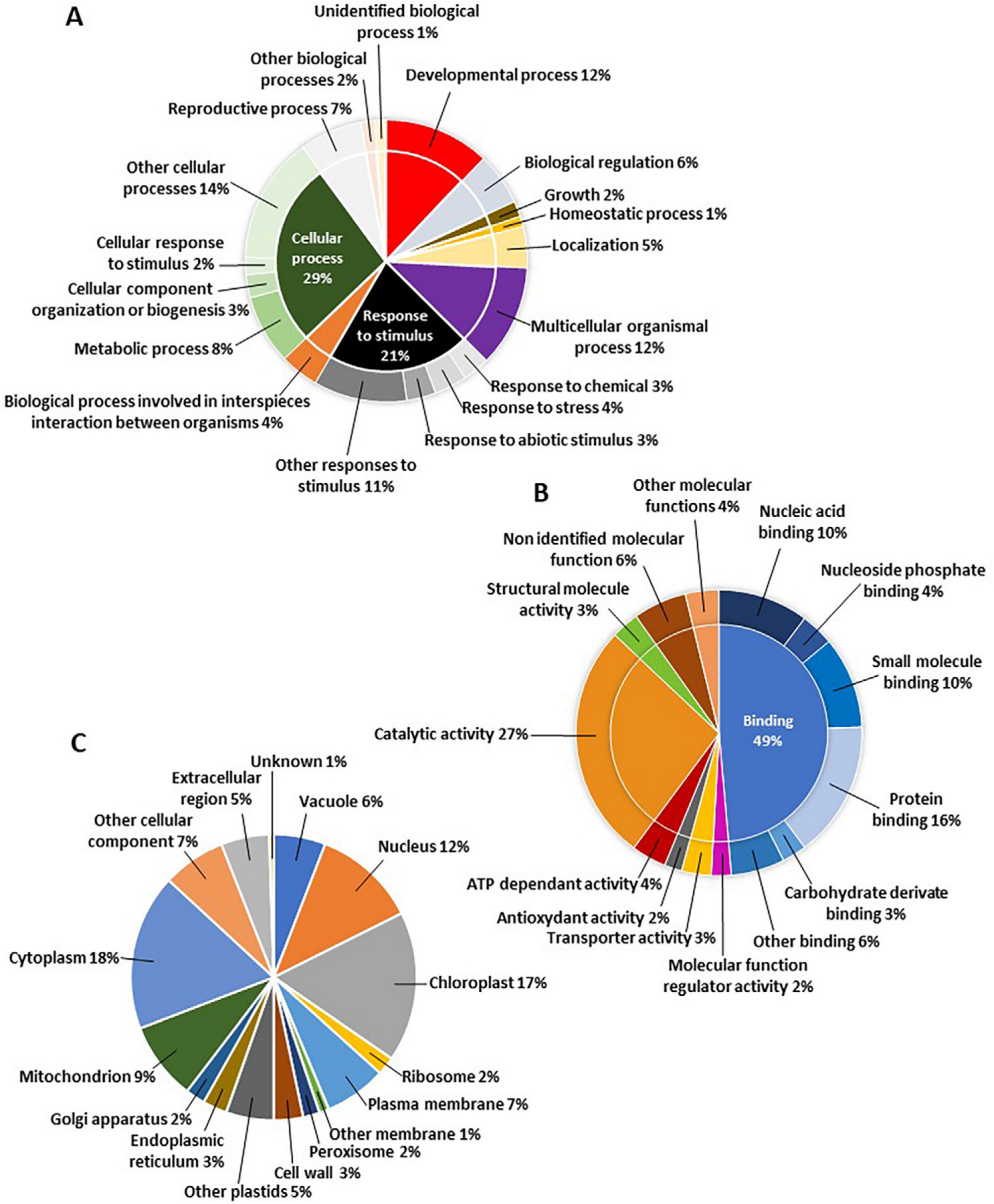
Diagram showing the biological processes (A), molecular functions (B) and cellular sites (C) of identified proteins in *Klebsormidium nitens.*

The data described here were obtained by analyzing the proteome of the alga *K. nitens* grown under control conditions or in the presence of 500 mM NaCl for 1 or 3 h. As algae grow slowly *in vitro*, our analysis was limited to two independent samples for each condition (control or NaCl stress). A total of 2324 and 2803 proteins were identified with the Proline software in the control and NaCl treatment conditions, respectively. The corresponding datasets are available *via* ProteomeXchange with identifier PXD066259. The identification and the GO terms associated with the proteins are given in the supplementary material (supplementary file 1). To increase the robustness of the bioinformatic analysis, the lists obtained were filtered to validate only proteins identified with at least 2 peptides and in the two samples from the same condition. A total of 1190 *K. nitens* proteins were identified and validated, of which 124 were found only under NaCl treatment and 62 only in the control condition (supplementary file 1). 922 of the 1190 proteins belong to orthologous groups and have been classified according to their cellular components, biological processes and molecular functions categories (Fig. 1).

**Fig. 2:**
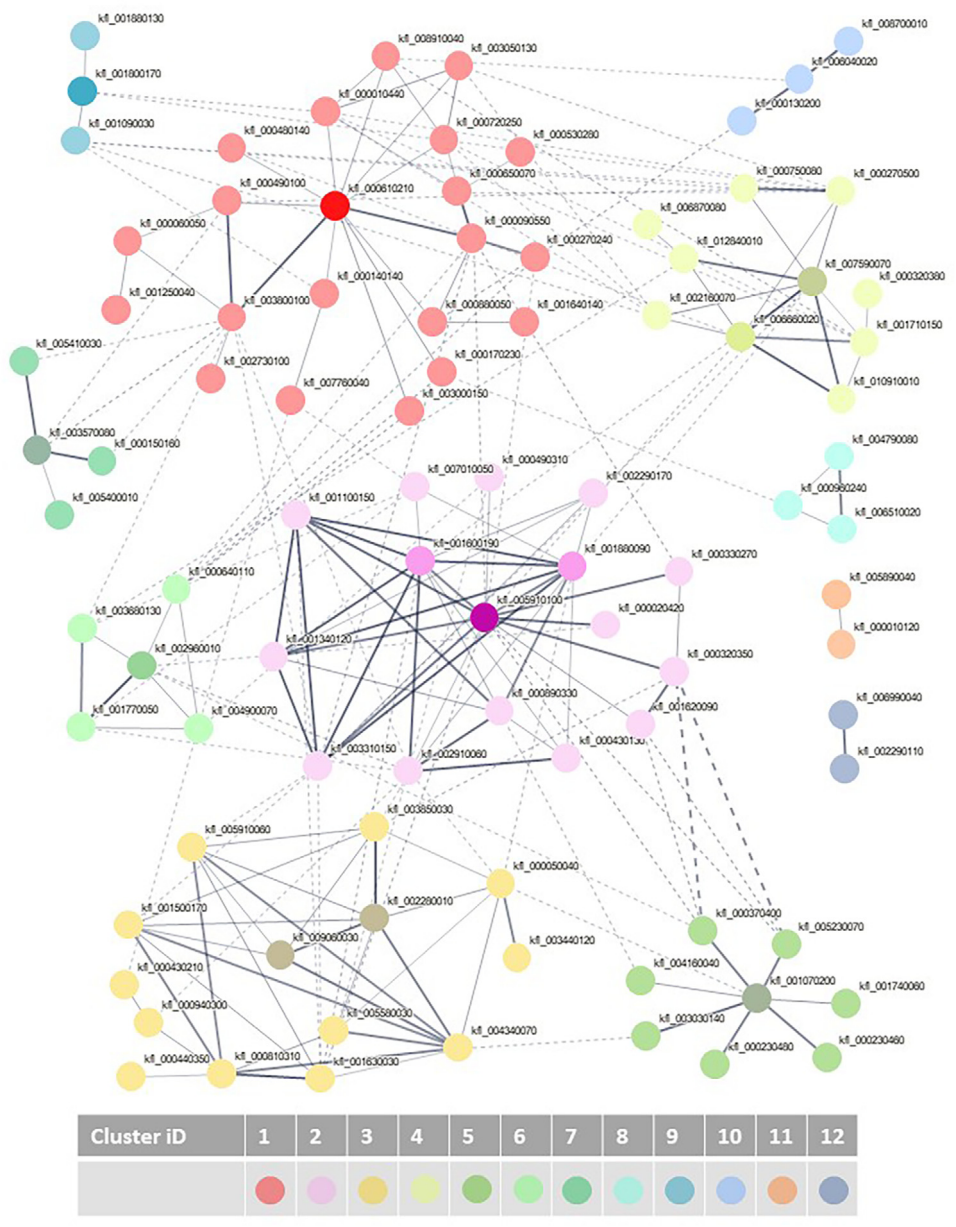
Protein-protein interaction network of the 124 *K. nitens* proteins detected specifically after NaCl treatment. The network was constructed with STRING v12. The proteins are represented by nodes whereas their interactions by edges (thickness indicate the level of confidence; minimum confidence = 0.4). Edges between clusters are in dotted lines. The twelve different clusters are identified by colors.

Proteins found only in saline conditions were analyzed using STRING (Search Tool for the Retrieval of Interacting Genes/Proteins) software version 12.0 [5]. This analysis enables us to distribute the 124 proteins into 12 clusters (Fig. 2). The proteins (identifier, *A. thaliana* orthologue, protein name) identified within each cluster are shown in supplementary file 2.

This analysis reveals key proteins that could help to understand how *K. nitens* responds to salt stress. A few clusters caught our attention.

Cluster 1 is composed of several proteins known to be involved in amino-acid synthesis. Among them, the protein kfl_000610210 (Tryptophan synthase β subunit 1 TSB1), essential for Trp synthesis in plants, clearly illustrates the link between amino acid synthesis and stress responses. Indeed, TSB1 was recently identified as a coordinator in the trade-off between plant growth and abiotic stress responses [6].

Proteins found in clusters 2, 3 and 5 are predicted to be involved in gene expression (transcription and translation). In cluster 2, five proteins are identified as ribosomal proteins or their precursor. Interestingly, kfl_005910100 (putatively RAD23), the most connected hub protein in this cluster, plays major roles in DNA repairing during abiotic stress (*e.g.* heat stress; [7]). In cluster 3, the two proteins that are the most connected, kfl_002280010 (small nuclear ribonucleoprotein G) and kfl_009060030 (SR1 orthologue in *A. thaliana,* supplementary file 1, sheet “total protein”) and their interactions with 12 other proteins reinforce the role of RNA -binding proteins and regulation mRNA processing in response to saline stress. Cluster 5 is composed of proteins involved in nucleosome assembly (chromatin remodeling) with kfl_001740060 orthologue to DEK domain-containing chromatin-associated protein 1 in *A. thaliana*, or histone H2A (kfl_001070200) occupying a central position. In addition to the regulation of gene expression and the synthesis of new proteins, clusters 6 and 11 include proteins involved in protein folding and turnover, respectively. Interestingly, the chaperonin kfl_002960010 (cluster 6) and kfl_000010120 (cluster 11) are proteins associated with loss of desiccation tolerance [8] and rehydration [9].

In cluster 4, the protein kfl_007590070 (enoyl-CoA hydratase, MFP2) interacts with several proteins involved in β oxidation of fatty acids (*e.g.* 3-ketoacyl-CoA thiolase), a catalytic pathway essential for a plethora of physiological processes in plants including senescence, germination or responses to biotic and abiotic stress [10,11].

Finally, the analysis has identified three clusters (8, 10 and 12) highlighting the crossover between abiotic stress and primary metabolism, and particularly carbohydrate metabolism. Cluster 12 contains glucose-6-phosphate 1-dehydrogenase and galactose mutarotase-like protein. Clusters 8 and 10 contain proteins involved in glycolysis and starch synthesis respectively, including phosphofructokinase, pyrophosphate–fructose-6-phosphate 1-phosphotransferase, fructose-6-phosphate 1-phosphotransferase, soluble starch synthase III-1 and starch synthase 2.

## 4 Experimental Design, Materials and Methods

### 4.1 K. nitens samples preparation

*K. nitens* strain SAG 13.91 (Department Experimental Phycology and Culture Collection of Algae, Göttingen, Germany) was grown in a modified liquid C medium (supplementary file 1 for composition) at 20 °C, under stirring (130 rpm) and 16 h photoperiod at 60 µmol.m^−2^. s^−1^. Two weeks old cultures were divided into four samples and equilibrated for 2 h under stirring and light. NaCl and l-arginine were added in two samples (final concentrations of 500 mM and 5 mM respectively). The two others (control samples) were grown with l-arginine but without NaCl. After one and three hours of treatment, *K. nitens* cells were harvested by vacuum on cellulose ester membrane of 0.2 µm pores and were immediately frozen in liquid nitrogen. Cells from the same initial sample and with the same treatment were pooled before further analysis. Frozen cells were mechanically ground using 5 mm diameter glass beads and a MM200 oscillating vibro-mill 72 (Retsch). The cell powder was mixed with a buffer made of Tris 50 mM pH7.5, NaCl 150 mM, EDTA 1 mM, NP40 1 %, glycerol 10 %, in presence of Protease Inhibitor Cocktail (Roche). In order to partially purify the protein fraction, 4 vol of 100 % acetone were added to the samples and incubated for 1 h at −80 °C. Then, the proteins were precipitated by centrifugation for 10 min at 14,000 g and washed four times with 80 % acetone. Finally, proteins were resuspended in Tris 100 mM pH 7.6 and the concentration of each sample was estimated using a nanodrop (Thermofisher). The protein concentration was adjusted to 2 µg/µl in Tris 100 mM pH 7.6 with 5 % SDS and 25 mM DTT.

### 4.2 S-Trap digestion and nano-LC-MS/MS

100 µg of proteins samples were reduced and alkylated using equilibration buffers containing dithiothreitol and iodoacetamide onto a S-Trap™ mini spin column (ProtiFi, NY USA), and digested by the addition of 125 *µ*L of a solution of modified trypsin in 50 mM ammonium bicarbonate (8 ng/µL, sequence grade, Promega). The mixture was incubated at 37 °C overnight. The peptides are eluted by 50 mM ammonium bicarbonate and then by 0.2 % formic acid and finally by 50 % aqueous acetonitrile containing 0.2 % formic acid. The pooled eluates are dried down and resuspended in 60 µL of 2 % acetonitrile, 0.05 % trifluoroacetic acid, vortexed and sonicated for 10 min before injection.

Peptides mixtures were analysed by nano-LC-MS/MS using nanoRS UHPLC system (Dionex ) coupled to an Q-Exactive Plus mass spectrometer (Thermo Fisher Scientific). Five microliters of each sample were loaded on a C18 pre-column (5 mm × 300 *µ*m; Thermo Fisher) at 20 *µ*L/min in 5 % acetonitrile, 0.05 % trifluoroacetic acid. After 5 min of desalting, the pre-column was switched on line with the analytical C18 column (15 cm × 75 µm; Reprosil C18 in-house packed) equilibrated in 95 % of solvant A (5 % acetonitrile + 0.2 % formic acid in water) and 5 % of solvant B (80 % acetonitrile + 0.2 % formic acid in water). Peptides were eluted using a 5–50 % gradient of B during 105 min at a 300 nL/min flow rate. The Q-Exactive Plus was operated in data-dependent acquisition mode with the Xcalibur software. Survey scan MS spectra were acquired in the Orbitrap on the 350–1500 *m/z* range with the resolution set to a value of 70 000. The ten most intense ions per survey scan were selected for HCD fragmentation, and the resulting fragments were analysed in the Orbitrap with the resolution set to a value of 17 500. Dynamic exclusion was used within 30 s to prevent repetitive selection of the same peptide.

### 4.3 Protein identification and quantification: database search and data analysis

Acquired MS and MSMS data were searched with Mascot (version 2.8.0.1, http://matrixscience.com) against a custom-made database containing all *K.nitens* proteins from the UniProtKB database (Swiss-Prot/TrEmbl release 20,240,521, 16,263 entries).

The search included methionine oxidation, N-ter acetylation as variables modifications and carbamidomethylation of cysteine as a fixed modification. Trypsin was chosen as the enzyme and 2 missed cleavages were allowed. The mass tolerance was set to 10 ppm for the precursor ion and to 20 mmu for fragment ions. Raw MS signal extraction of identified peptides was performed across different samples. Validation of identifications was performed through a false-discovery rate set to 1 % at protein and peptide-sequence match level, determined by target-decoy search using the in-house-developed software Proline software version 2.1 (http://proline.profiproteomics.fr/) [12].

The mass spectrometry proteomics data have been deposited to the ProteomeXchange Consortium *via* the PRIDE [13] partner repository with the dataset identifier PXD066259.

Overall, 1575 *K. nitens* proteins were identified in at least one sample. For further bioinformatic analyses, we retained the proteins identified in both control samples and/or in both samples treated with NaCl, *i.e.*, a total of 1190 proteins.

### 4.4 OrthoFinder analysis and computational processing

To enhance the functional annotation of *K. nitens* proteins, orthogroups were identified using the software OrthoFinder (version 3.0.1b1) [14] using default settings. The analysis was performed based on the proteomes of 13 plant species, including Brassicaceae (*Arabidopsis thaliana, Brassica juncea, Brassica napus, Brassica oleracea, Camelina sativa, Eutrema salsugineum*), Fabaceae (*Cajanus cajan, Glycine* max*, Lupinus angustifolius, Medicago truncatula, Pisum sativum, Trifolium pratense*), and the proteome deduced from *K. nitens* genome. The proteomes were retrieved from EnsemblPlants (release 56) February 2023 [15] for the rosids species, while the reference proteome of *K. nitens* (*Klebsormidium nitens* NIES-2285 v1.1) was obtained from [4].

### 4.5 Data processing and GO term assignment

The generated orthogroups were filtered to retain only those containing proteins from both *A. thaliana* and *K. nitens*. The Gene Ontology (GO) terms associated with *A. thaliana* proteins were then transferred to the corresponding *K. nitens* homologs within the same orthogroups.

The GO hierarchy file, available from the Gene Ontology Ressource [16,17,18] was used to map initial GO terms to their most relevant parent terms. This selection was performed manually for subcellular localization categories and semi-automatically for biological process and molecular function categories, using the R package ontology Index [19]. To avoid redundancy, only one instance per parent term was retained for each protein. GO annotations for *A. thaliana* were obtained from the TAIR database [20].

## Limitations

As algae grow slowly *in vitro*, we were limited by the number of samples sent for analysis (2 samples for each condition). The analysis was therefore limited to a qualitative level and did not allow a quantitative comparison of protein levels between the two conditions.

## Ethics Statement

The authors have read and follow the ethical requirements for publication in Data in Brief and confirming that the current work does not involve human subjects, animal experiments, or any data collected from social media platforms.

## Credit Author Statement

**Alix Martinet**: Conceptualization, Methodology, Data curation, Writing-original draft preparation, Writing-Reviewing and Editing. **Carole Pichereaux**: Conceptualization, Methodology, Data curation, Writing-original draft preparation, Writing-Reviewing and Editing. **David Wendehenne**: Reviewing and Editing, Fund raising, Administration. **Sylvain Jeandroz**: Conceptualization, Supervision, Writing-original draft preparation, Writing-Reviewing and Editing. **Valérie Nicolas-Francès:** Conceptualization, Supervision, Data curation, Writing-original draft preparation, Writing-Reviewing and Editing.

## Data Availability

Proteomic profile data of Klebsormidium nitens alga grown in control and saline conditions (Original data).

## Appendix Supplementary materials

Image, application 1

Image, application 2
